# Contrasting Spatial Patterns in Active-Fire and Fire-Suppressed Mediterranean Climate Old-Growth Mixed Conifer Forests

**DOI:** 10.1371/journal.pone.0088985

**Published:** 2014-02-20

**Authors:** Danny L. Fry, Scott L. Stephens, Brandon M. Collins, Malcolm P. North, Ernesto Franco-Vizcaíno, Samantha J. Gill

**Affiliations:** 1 Department of Environmental Science, Policy, and Management, University of California, Berkeley, California, United States of America; 2 USDA Forest Service, Pacific Southwest Research Station, Davis, California, United States of America; 3 University of California Center for Fire Research and Outreach, College of Natural Resources, Berkeley, California, United States of America; 4 Departamento de Biología de la Conservación, Centro de Investigación Científica y de Educación Superior de Ensenada, Ensenada, Baja California, México; 5 Natural Resources Management and Bioresource and Agricultural Engineering Departments, California Polytechnic State University, San Luis Obispo, California, United States of America; University of Missouri, United States of America

## Abstract

In Mediterranean environments in western North America, historic fire regimes in frequent-fire conifer forests are highly variable both temporally and spatially. This complexity influenced forest structure and spatial patterns, but some of this diversity has been lost due to anthropogenic disruption of ecosystem processes, including fire. Information from reference forest sites can help management efforts to restore forests conditions that may be more resilient to future changes in disturbance regimes and climate. In this study, we characterize tree spatial patterns using four-ha stem maps from four old-growth, Jeffrey pine-mixed conifer forests, two with active-fire regimes in northwestern Mexico and two that experienced fire exclusion in the southern Sierra Nevada. Most of the trees were in patches, averaging six to 11 trees per patch at 0.007 to 0.014 ha^−1^, and occupied 27–46% of the study areas. Average canopy gap sizes (0.04 ha) covering 11–20% of the area were not significantly different among sites. The putative main effects of fire exclusion were higher densities of single trees in smaller size classes, larger proportion of trees (≥56%) in large patches (≥10 trees), and decreases in spatial complexity. While a homogenization of forest structure has been a typical result from fire exclusion, some similarities in patch, single tree, and gap attributes were maintained at these sites. These within-stand descriptions provide spatially relevant benchmarks from which to manage for structural heterogeneity in frequent-fire forest types.

## Introduction

Mediterranean climate regions are botanically diverse, but also include intense human influences due to high population densities and intensive agriculture. Mediterranean-type ecosystems are found on the western edge of continents between 30–40 degrees north and south of the equator, and are characterized by summer drought, and large interannual variability in precipitation [1]. There are five Mediterranean climate regions in the world, three in the southern hemisphere (southwestern Australia, southwestern South Africa, and central Chile) and two in the northern hemisphere (Mediterranean Basin (MB), California - Northern Baja California (CNB)).

Because of the wet season accumulation of plant biomass, which then dries during the long dry season, vegetation in Mediterranean landscapes is among the most fire prone in the world [2]. The effect of fire on Mediterranean landscapes is receiving increased attention as climates warm and intensive human land-use increases [3], [4]. Most climate-change models predict that Mediterranean climate regions will experience longer and more frequent droughts, with greater interannual variability. Collectively, these effects are expected to lead to increased intensity and frequency of fire [5]–[8].

The two Mediterranean climate regions in the northern hemisphere have experienced differing land use histories. Many cultures have evolved in the MB (e.g. Mesopotamian, Egyptian, Phoenician, Hebrew, Greek, Arab, Roman), with many political conflicts (wars, changes in land ownership, migrations) that generated numerous socioeconomic and land-use changes [3]. Millennia of intensive use including burning, cutting and grazing of non-arable lands, and clearing, terracing, and cultivation of arable areas have resulted in strongly human-modified landscapes. Consequently, most of these ecosystems currently exist outside their natural states [4]. Given the extensive ecosystem changes in MB landscapes, areas that can be considered to be in a relatively natural state are extremely rare [9]. In fact, Keeley et al. [4] suggests that no natural reference landscapes exist in the contemporary MB climate region, an area of over 1,000,000 square km.

In contrast, CNB has experienced a much different land use history. Although agriculture flourished in parts of the Americas, it was absent in the California’s until late in the eighteenth century when Spaniards first settled the CNB coastline [4]. Prior to that, it was inhabited for millennia by aboriginal peoples, who managed ecosystems for diverse purposes, with fire being their primary tool [10]. Compared with other Mediterranean climate regions in the world, the landscapes in CNB and Chile have had the shortest period of intensive human land management [4].

In California and other parts of the western US, regimes of frequent, low to moderate intensity fires were once a fundamental component of many pine-dominated ecosystems [11]–[16]. However, in the western US fire has been essentially excluded for a century or more, resulting in substantially altered forest conditions [17]–[19]. Forests have become denser, primarily in smaller tree-size classes, and species compositions have shifted toward shade-tolerant trees, which are less adapted to frequent fire [20]. This altered condition exists in drier forests throughout the western U.S. Consequently, most information on forest dynamics is available for areas that were harvested or have been under a policy of fire exclusion for the last century [21]. Studies are lacking, however, for reference forests that are still affected by contemporary climate and natural disturbance regimes.

The majority of California Jeffrey pine (*Pinus jeffreyi* Balf.) forests were harvested in the late 19^th^ or early 20^th^ centuries to support mining operations [22], [23]. Fire suppression was initiated shortly after or coincident with these harvests. Presently Jeffrey pine forests in the Sierra Nevada are burning at much greater proportions of high-severity than what occurred historically [24]; additional areas of Jeffrey pine and mixed conifer forests with high fire hazards occur in the southern California mountains. The Jeffrey pine dominated forests in the Sierra San Pedro Mártir (SSPM), northern Baja California, Mexico, have not experienced widespread fire suppression or harvesting [25]. Fire return intervals in the SSPM forests, while variable over the past few centuries, were influenced by interactions with climate [26] and anthropogenic and lightning ignition patterns [27] and have remained short into the mid- to late 20^th^ century [25], [28]. The SSPM therefore offers an opportunity to describe stand structures, composition, and dynamics in forests that still experience wildfire. Indeed the forested ecosystems in northern Baja California are probably the only extant large, Mediterranean-climate reference forests in the northern hemisphere.

Old-growth reference sites provide information on ‘desired’ forest conditions [29], either as contemporary forests with functioning fire regimes (e.g., [28], [30]) or as near pre-Euro/American settlement forests reconstructed from historical data (e.g., [18], [19], [23], [31], [32]). They contain complex structures and spatial patterns indicative of resilient forests, with the ability to maintain ecosystem functions while incorporating disturbances of diverse types (e.g., [28], [33], [34]). Recent studies have focused on describing patterns within the stand as three main components: clusters or patches of trees, large solitary trees, and canopy gaps (those reviewed in [35]–[37]). Yet, as reflected in the heterogeneity captured in the limited number of studies available, there is still a need for this type of information from reference sites and approaches to incorporating these characteristics into forest restoration prescriptions [36]. While the few remaining reference sites in the western US still have characteristics of old-growth forests (old and large trees, coarse downed wood, decadent snags [38], [39]), many are fragmented and have not burned in over a century. Since fire is one of the most important drivers of structural patterns, it is unclear how old-growth characteristics may have changed due to the exceptionally long fire-free period [30], [37], [40], and therefore their own need for restoration [41].

In this study, we compare patterns of forest structure in four old-growth Jeffrey pine-mixed conifer forest sites in Sierra Nevada, California USA and northern Baja California, Mexico. The sites compared capture a range of fire history, climate, and edaphic conditions, allowing for the potential identification of the different environmental influences on forest structure. Specifically, we characterize 1) spatial distributions of live trees among life history stages and snags; and 2) within-stand structural components: patches of trees, gaps, and widely spaced single trees. Given the difference in fire suppression policies, climate, and soils, the structural patterns are expected to be very different between the Sierra Nevada and Baja California sites. Our hypotheses are that the Sierra Nevada forests will have higher tree densities, fewer and smaller canopy gaps, and less spatial variability, primarily due to cessation of fire for over a century. In contrast, the forests in northern Baja California are expected to have more complex spatial structure and canopy gap characteristics. Information from this study could assist in further defining desired conditions for restoration treatments in similar forests in the western US [29], [38], [42], [43] and possibly other pine dominated forests in the MB that are adapted to frequent, low-moderate intensity fire regimes.

## Methods

### Study Areas

**Figure 1:**
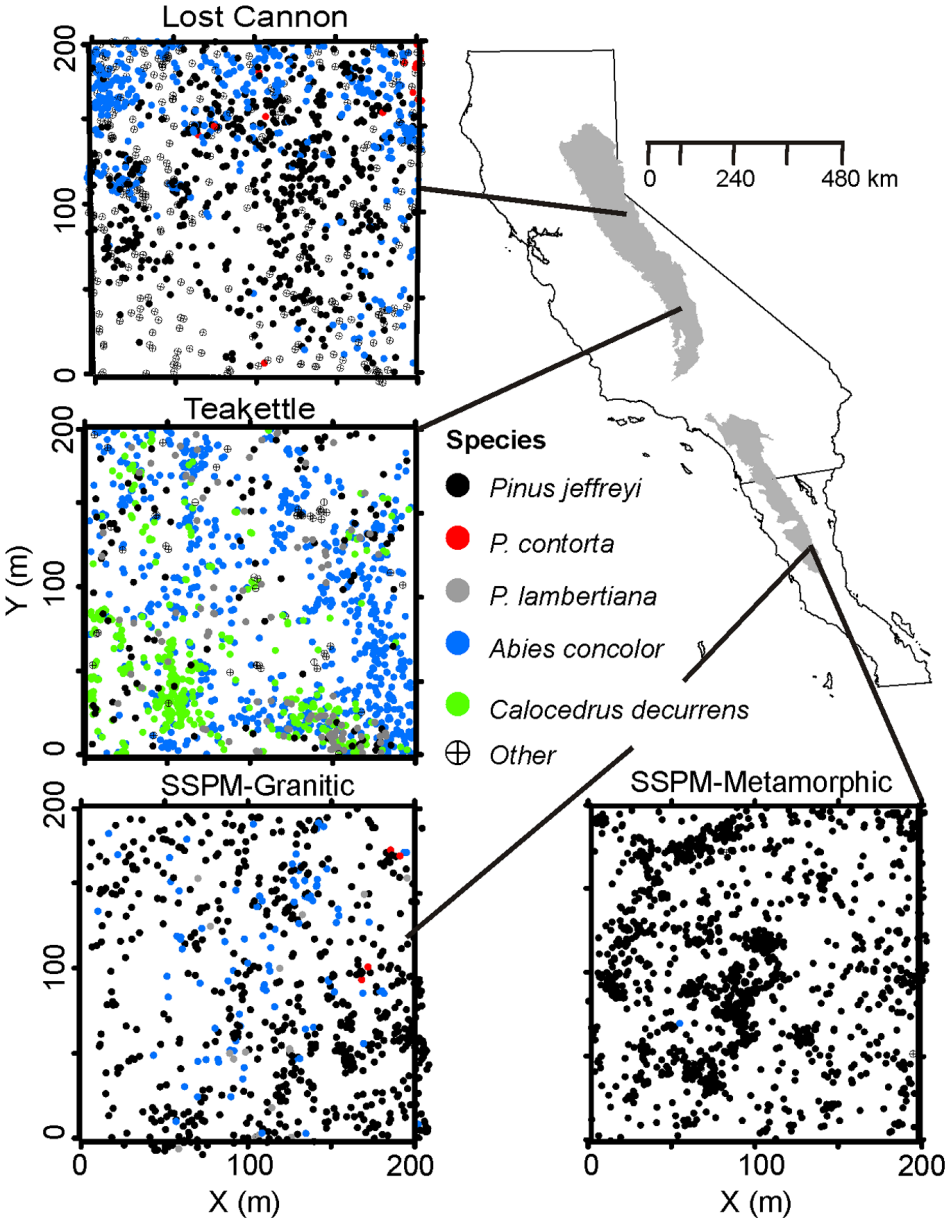
Study sites and live tree (diameters >5 cm) locations in old-growth Jeffrey pine-mixed conifer forests in the Sierra Nevada (upper shaded area), eastern California, and the southern Peninsular Range (lower shaded area), northwestern Mexico. Species identified as other include *Juniperus occidentalis*, *Abies magnifica*, *Populus tremuloides*, *Prunus emarginata*, *Quercus kelloggii*, and *Q. chrysolepis*.

The Sierra Nevada sites are in the Lost Cannon Creek watershed, which is in the central portion of the range on the eastern slope approximately 30 km northwest of Bridgeport (Humboldt-Toiyabe National Forest: 38° 24′ N, 119° 28′ W), and Teakettle Experimental Forest, which is in the southern portion of the range on the western slope (Sierra National Forest: 36° 58′ N, 119° 02′ W) (Figure 1). The Baja California sites are in the Sierra San Pedro Mártir National Park (SSPM; 31° 37′ N, 115° 59′ W), located on the southern Peninsular Range in north-central Baja California, Mexico (Figure 1). All sites have never been harvested for timber, but have a history of livestock grazing at varying intensities. Commencing in the early 19^th^ century, cattle and sheep supported the San Pedro Mártir Mission in the SSPM [25], [44], and in the 19^th^ century, cattle and sheep were grazing in the Sierra Nevada [45].

Study areas are within the North American Mediterranean climate zone, although the North American Monsoon System may influence the SSPM [26]. There is no long-term climate information from the SSPM study areas. The closest long term weather data comes from the Santa Cruz Station (980 m above sea level; 1960–2004) where mean annual precipitation is 33 cm, 13.7% of which falls during the summer months (June through September). Mean annual summer and winter temperatures are 25°C and 12°C, respectively. Annual precipitation measured with temporary weather station on the northern plateau (2400 m; 1989–1992) in the SSPM, 1.2 km east and south of the study areas, was 55 cm [46] with ∼28% falling in summer [47]. For Lost Cannon, mean annual precipitation at the closest weather station (Bridgeport) is 25.4 cm, 12.2% of which falls during the summer. Mean annual summer and winter temperatures are 15°C and 3°C, respectively. Mean annual precipitation at Teakettle is 125 cm, with only 2.7% falling during the summer. Mean annual summer and winter temperatures are 15.5° and 0.7°C, respectively [48].

While Jeffrey pine is present at all sites, edaphic conditions and disturbance history vary and consequently species compositions are distinguishing characteristics. Lost Cannon (2500 m) is a Jeffrey pine-mixed conifer forest on soils derived from weakly developed decomposed granite. Soils are shallow typic cryo-xeropsamments of loamy coarse sand. *Populus tremuloides* (Michx.) is common on more mesic, lower slopes. The SSPM granitic site (SSPM-Gran; 2410 m) is also a Jeffrey pine-mixed conifer forest on soils derived from decomposed granite. Soils are typic xeropsamments, mostly loamy sands. Chemical and textural properties reported in [49] are similar to typical granite-derived soils in comparable forests in California [50]. The SSPM metamorphic site (SSPM-Meta; 2440 m), located approximately 2 km north of SSPM-Gran, is a monotypic stand of Jeffrey pine, with a small component of canyon (*Quercus chrysolepis* Liebm.) and Pacific emory oaks (*Q. emoryi* Torr.) in the understory. Soils are shallow, derived from metamorphic quartz schist and are sandy loams. Teakettle (2130 m) is an upper elevation mixed-conifer forest on the western slopes of the Sierra Nevada; it has the highest moisture regime of the four sites and is dominated by white fir (*Abies concolor* (Gord. & Glend.) Lindl.). Hardwoods in this forest include *Q. kelloggii* (Newberry) and *Prunus emarginata* ([Dougl. Ex Hook.] D. Dietr.). Soils are well-drained, mixed, frigid dystric xeropsamments, formed from decomposed granite [51].

Fire has been an important ecological component in these forests, occurring relatively frequently (fire return intervals of 4–24 years; [25], [45], [51]) prior to their respective fire cessation periods. Prior to 20^th^ century fire suppression policies in California [52], early sawmill and mining operations in the Sierra Nevada may have initiated the current fire-free period at Lost Cannon and Teakettle [23], [45], [51]. At the SSPM, road construction, land use changes, and limited summer fire suppression efforts have increased fire intervals in most areas of the forest commencing in the late 1960s [26], [46]; before this period systematic fire suppression did not occur in this area.

This research was carried out under Mexican Ministry of the Environment (SEMARNAT) permits NUM/SGPA/DGVS/3315, NUM/SGPA/DGVS/04036 and NUM/SGPA/DGVS/04075. We examined forest structure and spatial patterns in four-ha old-growth stem mapped plots and discuss possible mechanisms for any differences identified among sites. A four ha plot was selected because it should include a total of 1000–1500 trees, incorporating most of the spatial heterogeneity of these forest types [35]. Previous studies indicate pine-dominated forests that once experienced frequent, low-moderate intensity fires regenerated in small openings (e.g., [32], [53]). These openings were probably created when fires burned through local patches of recent tree mortality created by insects, disease, and drought [53]. Each 4 ha plot was established on a uniform slope aspect with the corner located randomly. The location (X and Y coordinates) of all stems (diameters >5 cm) by species were recorded using a high resolution global positioning system (GPS), and size attributes (diameter at breast height (DBH), height, and height to live crown base) were measured. We examined forest structure at three spatial scales: stand-level distribution patterns, within-stand canopy gaps and patches or clumps of trees, and individual trees. Variability in these three main structural components is recognized as a defining characteristic of resilient, frequent-fire conifer forests (reviewed in [35]).

### Fire History

Dendrochronology-based historical wildfire summaries have already been published for larger areas that included each of the study sites, Stephens et al. [25] for both SSPM sites, and North et al. [45], [51] for Teakettle and Lost Cannon, respectively. To characterize the fire history specifically for the stem mapped plots we selected a subsample of the original collection of fire-scarred trees, snags, and logs that were inside and within 100 m of the 4 ha plot perimeters. We calculated fire return intervals at two composite scales or filters using methods described in the abovementioned studies: all fire scars that were identified in the tree-ring record, and years in which a minimum of three samples and more than 25% of the recording samples were scarred. The non-parametric Kruskal-Wallis test was used to determine if significant differences (*p*<0.05) exist between sites at both composites (all fires and 25% filter). If an overall significant difference was identified, the Nemenyi test (non-parametric Tukey multiple comparisons test) was used to determine which sites differed (*p*<0.05, [54]).

### Spatial Patterns

To analyze snag and live-tree spatial distributions we used the pair correlation function *g*(*r*) (PCF), which is the derivative of the Ripley’s K-function and operationally similar. The PCF is recommended for identifying specific scales of deviation from a null model of complete spatial randomness (CSR) because of its non-cumulative properties [55], [56]. For trees that are independently and randomly distributed the PCF yields *g*_1_(*r*) = 1, under aggregation *g*_1_(*r*) >1, and under regularity *g*_1_(*r*) <1. Similarly, interactions among life history stages can be examined by extending the univariate analysis to the bivariate condition using the null hypothesis of independence and random labelling [56] among trees in different size classes and live trees and snags, respectively. Both univariate and pairwise patterns for life history stages of trees were analysed using the following size classes: small (DBH <25 cm), medium (DBH = 25–74 cm), and large (DBH ≥75 cm). We assume that the majority of trees in the small size class were established after fire cessation commenced by the 19^th^ century at Lost Cannon and Teakettle, and in contrast to the SSPM sites where fires continued well into the 20^th^ century. The PCF *g_12_(r)* is the observed densities of type 1 trees relative to type 2 trees; type 1 trees are independently and randomly distributed relative to type 2 trees if *g*_12_(*r*) = 1, positive interaction if g*_12_(*r*)* >1, and negative association if *g*_12_(*r*) <1 [56]. Observations of stem maps (Figure 1) indicate heterogeneity in tree distributions may be present. We evaluated univariate distributions of established trees (DBH >25 cm) to test the hypothesis of environmental homogeneity, and the need for inhomogeneous functions for the spatial analysis (see Methods S1).

All functions were computed up to a maximum of 50 m using an isotropic adjustment to correct for edge effects. To test the statistical significance (α = 0.05) of departure from null hypotheses for both univariate and bivariate observed *g (r)* we used the fifth maximum and minimum values of 199 Monte Carlo simulations as the test statistic [55]. If *g (r)* remains within the upper and lower approximately 95% simulation envelopes for any given *r* the null hypothesis is not rejected [56]. For the bivariate analyses the 95% confidence interval is calculated by holding locations of one population constant while using random toroidal shifts of the second population. Both *g_12_(r)* and *g_21_(r)* were evaluated for differences because we did not assume the interaction would be symmetric. We tested the overall departure from the null model at 0–50 m using the Goodness of Fit test statistic (GoF; [57]). Spatial analyses were implemented using the *spatstat* package (v. 1.22-1, [58]) in the R software environment [59].

### Forest Patch, Gap, and Single Tree Patterns

We further described within stand patterns using a method described in Plotkin et al. [60], which enables a summarization of among- and within-tree patch attributes such as density, proportion of trees in patches and patch size, density, basal area, and tree size distributions. The first step is to define a patch as two or more trees that are within a specified distance. Other studies have used this method with a range of intertree distances and examined the corresponding change in patch attributes [35], [61], [62]. Similar to Lydersen et al. [37], we use an intertree distance based on crown radii; two or more trees with overlapping crowns constitute a patch and projected crown area of the patch is then calculated. We estimated tree crown radius using species-specific equations developed from [63]. Crown width (*CW*) was estimated using the following:
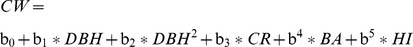
where coefficients b_0_ through b_5_ were developed from regression models using measurements from a network of forest plots in eight western US states. The Hopkins bioclimatic index (HI) is a geographic region variable and is estimated using the following:



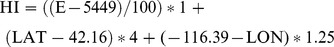
where E is the elevation, and LAT and LON is the latitude and longitude, respectively. Stem diameter (DBH), crown ratio (CR), basal area (*BA*), and *HI* were included in the model only if they were statistically significant. Model R^2^ values ranged from 0.61 to 0.85 for the softwoods and 0.45 to 0.59 for the hardwoods [63]. Patches were delineated using crown radius derived from CW in ArcGIS v.10 as described in Churchill et al. [36]. We controlled for edge effects by applying a 5 m buffer on the inside perimeter of the plot. This buffer eliminated the effect of tree crowns from trees outside the plot. To permit comparisons among sites and with other studies, we summarized structural attributes of tree patches into categories of 2–4, 5–9, and 10 or more trees per patch.

Very few studies have quantified gap characteristics due to shape complexity and clearly defined boundaries, and there is no standard method in delineating gaps with stem mapped data (but see [36], [37], [64]). Using the mapped tree patches identified above, we employed the Patch-Morph patch delineation algorithm [65] to identify contiguous gaps in the forest canopy. The Patch-Morph tool can be used in ArcGIS v.10, and while its intended use is for identification of habitat (e.g., wildlife patches and corridors) within areas of unsuitable habitat, we used this tool for a different, albeit parallel characterization. Users can specify two main parameters: gap maximum thickness (gap threshold) and patch minimum thickness (spur threshold). For this analysis, we chose a maximum gap threshold of 2 m, and a minimum spur threshold of 12 m, which is consistent with the gap delineation criteria used by Lydersen et al. [37]. We recognize that these values are somewhat arbitrary, however, they were selected by visually analyzing a range of gap and spur threshold combinations. Lower gap thresholds would identify only the smallest trees (crown radius less than 1 m) within mainly treeless areas resulting in highly complex shapes containing several ‘holes’ (or trees) within individual gaps, and larger spur thresholds failed to capture inherent shape heterogeneity. To compare the influence of edge effects– gaps may extend beyond the plot boundary– on canopy gap characteristics we report summaries with and without gaps where more than 10% of the gap perimeter overlapped the 5 m buffer.

Finally, to characterize the variation in tree patches within and among sites, we employed hierarchical cluster analysis. This technique classifies patches into mutually exclusive groups maximizing within-group similarity while minimizing between-group similarity [66]. The output is a dendrogram, or cluster tree, depicting the agglomeration sequence and the degree of similarity between groups containing like patches. We used Euclidean distances and Ward’s linkage method with the *hclust* function of the R statistical software [59]. The height along the cluster tree, and consequently the number of groups identified at that value, was chosen by using the following criteria: 1) observations of the patch distribution patterns indicated 3 to 6 natural groupings, depending on the level of scrutiny, and 2) after the initial clustering of patches and increasing stability (i.e. branch length) in the tree was evident. To identify important variables in distinguishing patch types we used principal components analysis (PCA) with the following inputs: mean, maximum, and standard deviation of tree diameters; trees per patch, tree density, patch area and patch perimeter. PCA was performed with the *prcomp* function of the R statistical software [59]. We used separate one-way analysis of variance (ANOVA) to test for differences in patch, gap, solitary tree, and patch group attributes among sites. If differences were detected (α <0.05), a post hoc Bonferroni means comparison test was used to determine which sites differed from each other. If any of the data did not meet the assumptions of normality or equal variance, we used a log_10_ transformation [54].

## Results

### Forest Structure

**Figure 2:**
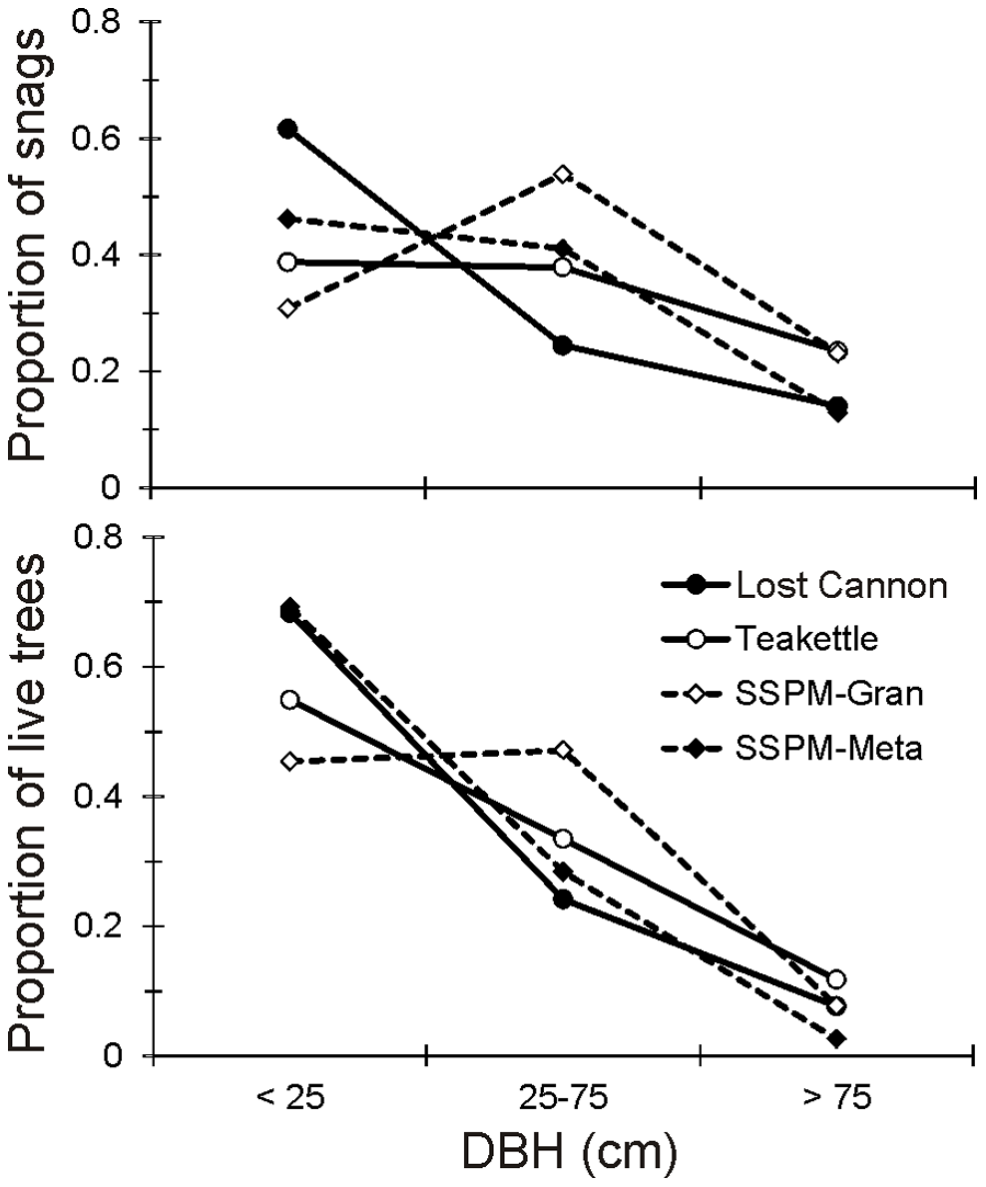
Proportion of trees within separate size classes in four old-growth Jeffrey pine-mixed conifer forest sites.

Jeffrey pine was the most common overstory species (Table 1, Figure 1), accounting for 64% to 99% of total basal area at both SSPM sites and Lost Cannon. At Teakettle white fir accounted for 47% of basal area while Jeffrey pine was 23%; but here Jeffrey pine had the largest mean DBH (63.0 cm, one standard error of the mean (SE) = 4.0 cm) followed by sugar pine (42.2 cm, SE = 3.9 cm) and white fir (35.0 cm, SE = 1.1 cm). Mean DBH of Jeffrey pine was similar at Lost Cannon (30.9, SE = 1.4 cm) and SSPM-Gran (33.0, SE = 0.9 cm). Mature Jeffrey pine trees were 20% smaller at SSPM-Meta compared to SSPM-Gran. Live tree and snag size distributions were skewed towards the smaller size classes (Figure 2). The largest trees (DBH ≥100 cm) accounted for 0.2% to 6% of the total number of live trees and 2.6% to 17% of the total number of snags.

**Table 1:** Site and average forest characteristics from four old-growth Jeffrey pine-mixed conifer forests in the Sierra Nevada, California, and northwestern Mexico.

Site	Elev. (m)	Type	Species Composition (%) (stems >5 cm DBH)	Density (stems ha^−1^)	
				Live	Snag
Lost Cannon	2500	Granitic	JP: 44.4, WF: 29.7, SJ: 18.1, QA: 5.7, LP: 1.3	337.8	21.5
Teakettle	2130	Granitic	WF: 52.1, IC: 28.0, JP: 8.5, SP: 7.8, QU: 1.6	346.3	27.8
SSPM-Gran	2410	Granitic	JP: 84.4, WF: 12.5, SP: 2.3, LP: 0.8	185.5	3.5
SSPM-Meta	2440	Metamorphic	JP: 99.5, QU: 0.4, WF: 0.1	345.5	9.8

SSPM, Sierra San Pedro Martir.

JP, *Pinus jeffreyi*; SJ, *Juniperus occidentalis* (Hook.); WF, *Abies concolor*; SP, *P. lambertiana* (Dougl. Ex. Loud.); QA, *Populus tremuloides*; QU, *Quercus* spp.; LP, *P. contorta var. murrayana* (Dougl. Ex. Loud.); IC, *Calocedrus decurrens* (Torr. (Florin)).

### Fire History

For all four sites, fire return intervals (FRI) were frequent prior to the onset of fire suppression (Table 2). Median FRIs ranged from 6 to 9 years for all fires, and 10 to 18 years for the 25% filter, which removes smaller fires that scar only one or two trees. The FRIs for the four sites were not significantly different for all fires (Kruskal-Wallis = 3.675, p = 0.299) or the 25% filter (Kruskal-Wallis = 5.606, p = 0.132).

**Table 2:** Mean (one standard deviation) fire return interval (FRI) summaries for old-growth Jeffrey pine-mixed-conifer forests in the Sierra Nevada, California, and northwestern Mexico.

	Lost Cannon	Teakettle	SSPM-Gran	SSPM-Meta
Time Period	1725–1900	1725–1900	1625–1970	1625–1970
All Fires FRI (yrs)	6.8 (3.8)	9.8 (7.5)	8.7 (7.0)	11.5 (9.0)
25% FRI (yrs)*	13.6 (13.1)	19.2 (9.0)	18.8 (10.2)	21.2 (11.7)
Last large fire	1896	1865	1946	1946

*Intervals calculated from fires that scarred three or more trees and at least 25% of the recording samples.

### Spatial Patterns

We compared the spatial distribution of trees >25 cm DBH to the null model of CSR for all four sites to determine if large-scale environmental heterogeneity was present, which affect spatial pattern interpretations (Figure S1). Significant aggregation was apparent for Lost Cannon and Teakettle but not at the SSPM sites. To adjust for this, in subsequent spatial analyses we used inhomogeneous and homogeneous PCFs for the Sierra Nevada and SSPM sites, respectively.

**Figure 3:**
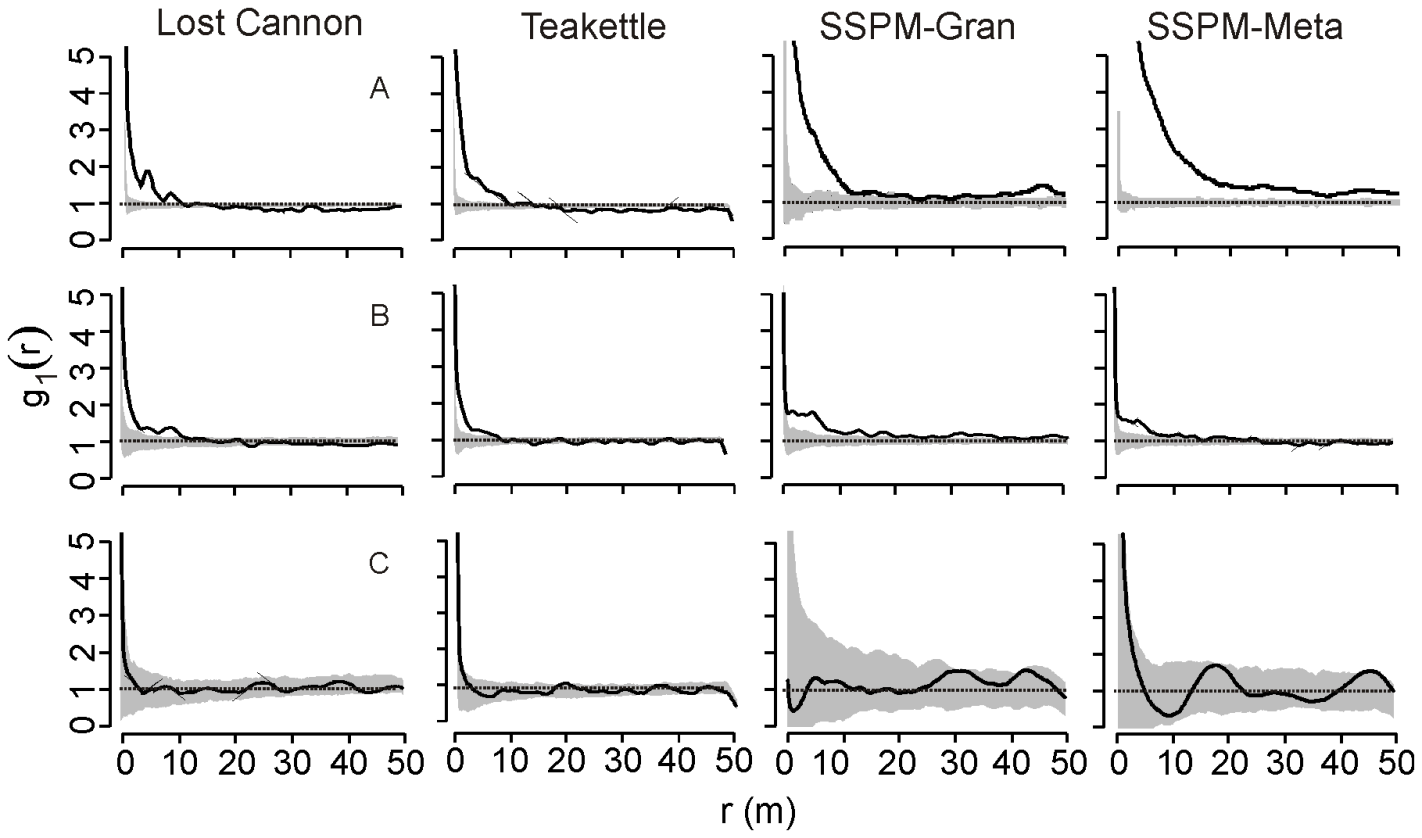
Comparison of the spatial pattern of live trees from four old-growth Jeffrey pine-mixed conifer forests (solid lines). The inhomogeneous pair correlation function with an Epanechnikov smoothing kernel was used for Lost Cannon and Teakettle. Analysis of tree size classes are arranged in rows: A, 5–25 cm DBH; B, 25–50 cm; C, 50–75 cm; and D, DBH >75 cm. Approximately 95% simulation envelopes (grey shaded areas) were constructed with 199 Monte Carlo simulations of the CSR model.

We used the live tree size-classes in Fig. 2 to analyze spatial patterns, and graphical distributions showed evidence of significant departure from random: the strength and range of clustering declined systematically with increasing tree size. Small (5–24 cm DBH) to mid-sized (25–74 cm DBH) trees were most strongly aggregated at scales less than 20 m (GoF p-value <0.005; Figure 3A & 3B). There was stronger evidence of clustering at the SSPM sites compared to Lost Cannon and Teakettle. The largest trees (>75 cm DBH) were not significantly different from random (GoF p-value >0.10; Figure 3C).

**Figure 4:**
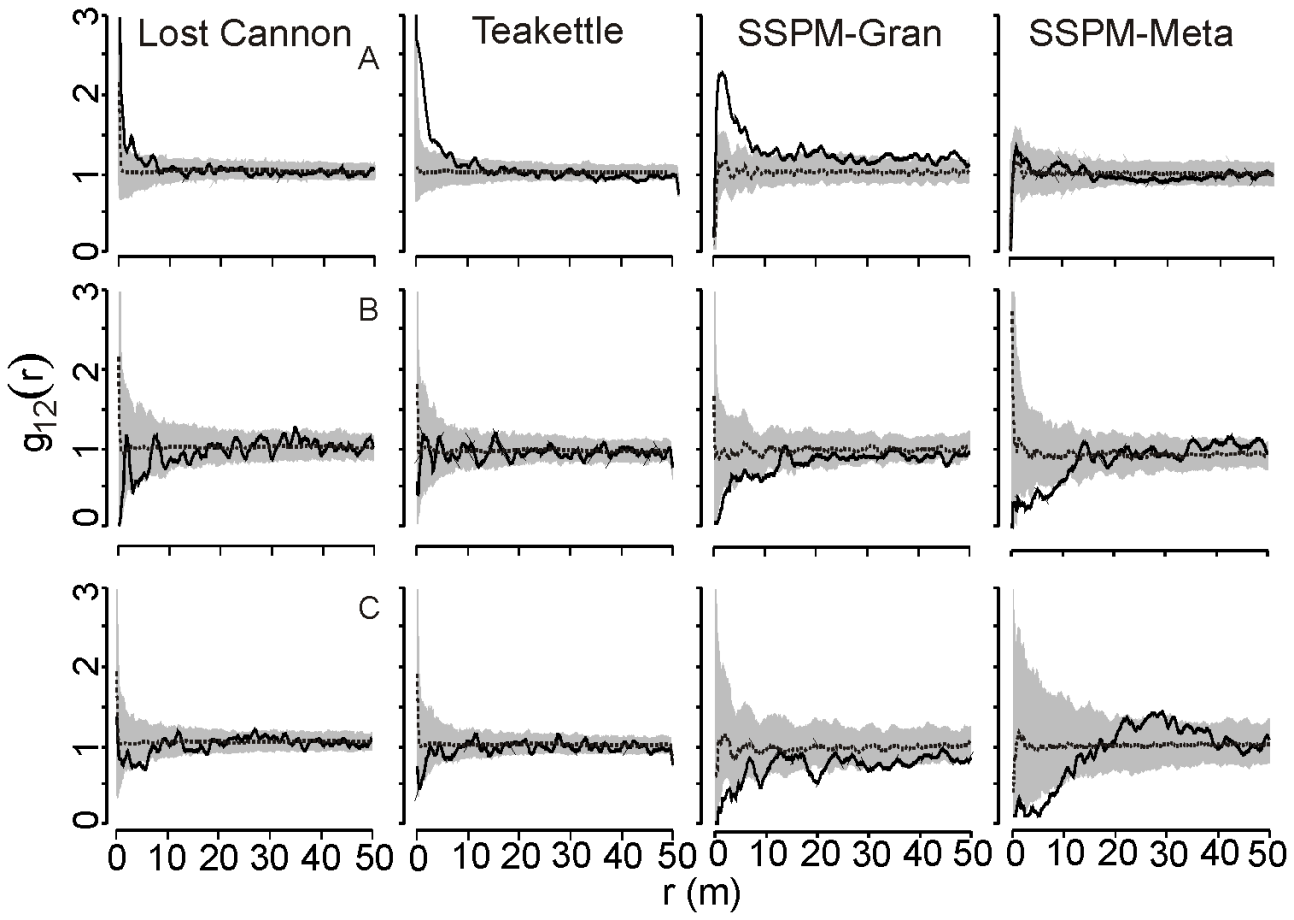
Bivariate pair correlation functions for all pairwise tree size classes (solid lines) from old-growth Jeffrey pine-mixed conifer forests. Size class comparisons are arranged in rows: A, 5–24 and 25–74 cm DBH; B, 25–74 cm and ≥75 cm DBH; and C, 5–24 cm and ≥75 cm DBH. The inhomogeneous pair correlation function with an Epanechnikov smoothing kernel was used for Lost Cannon and Teakettle. Approximately 95% Monte Carlo simulation envelopes (grey shaded areas) were constructed with the null model of independence.

Bivariate PCF for independence of small and medium trees showed strong evidence for small-scale association at SSPM-Gran, Lost Cannon, and Teakettle (GoF p-value <0.005; Figure 4A). Comparing small and medium with large trees, distributions were not significantly different from independent for all sites (GoF p-value >0.05) except SSPM-Meta, which showed a negative association of small to large trees (GoF p-value <0.05; Figure 4C).

**Figure 5:**
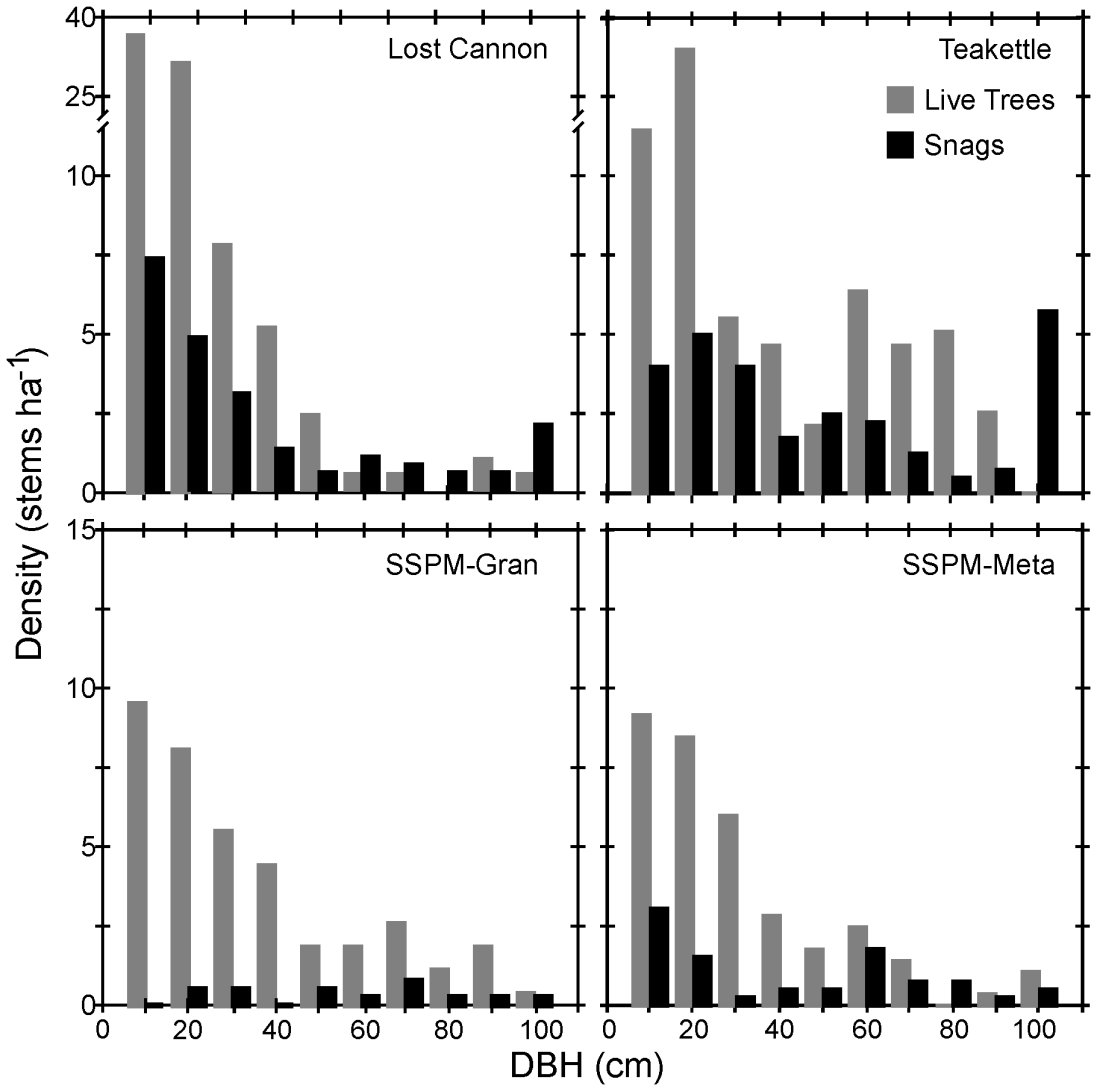
Tree diameter distributions (10 cm size classes) by density for snags and live single (i.e., non-patch) trees in 4-ha stem mapped plots.

**Figure 6:**
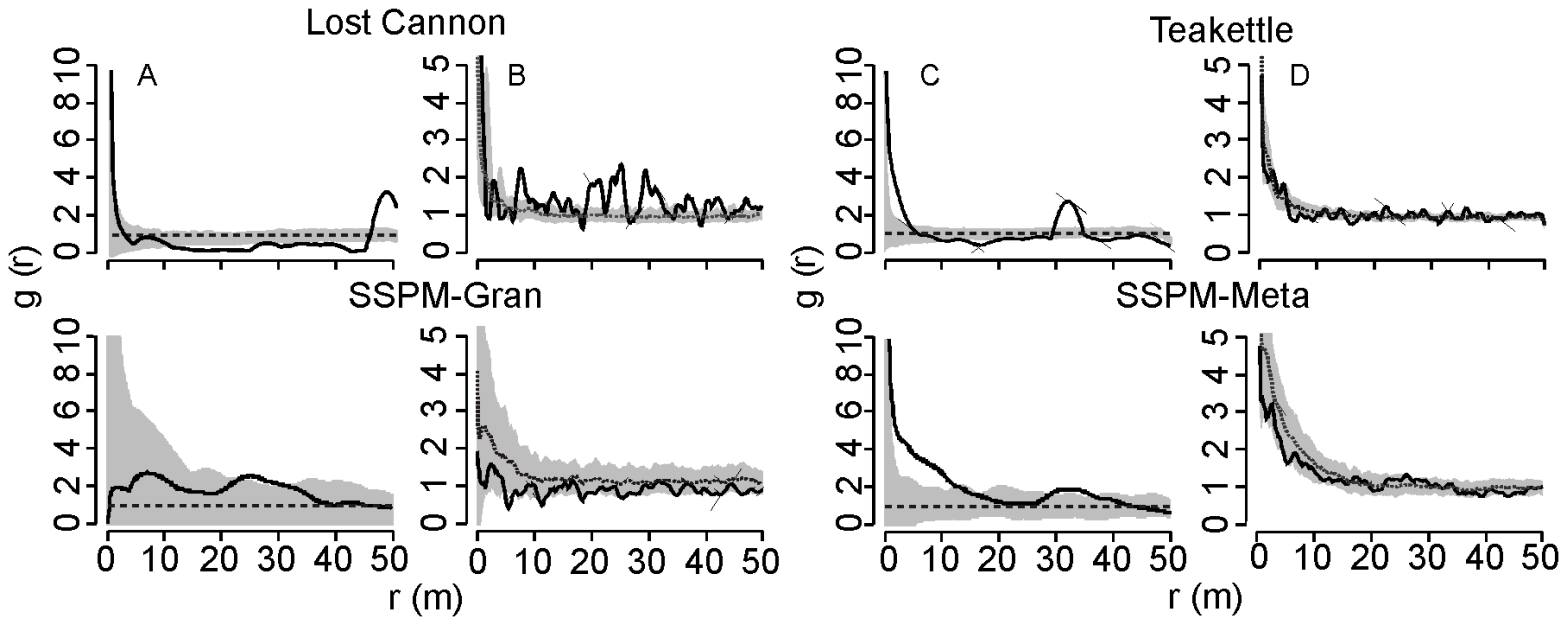
Comparison of the observed (solid lines) spatial distribution of snags (A and C) and snags with live trees (B and D). The inhomogeneous pair correlation function with an Epanechnikov smoothing kernel was used for Lost Cannon and Teakettle. Approximately 95% Monte Carlo simulation envelopes (grey shaded areas) were constructed with the null model of random mortality.

Snag size distributions were skewed towards the smaller size classes, especially for Lost Cannon and Teakettle, although most of the largest snags (DBH >100 cm) were also from these sites (Figure 5). There was an overall significant difference in mean snag size among the four sites (p-value <0.000), with Lost Cannon (31.8 cm, SE = 3.9 cm) being smaller than SSPM-Gran (54.0 cm, SE = 8.0 cm) and Teakettle (50.7 cm, SE = 4.2 cm). Analyzing all snag size classes, there was evidence of a nonrandom spatial distribution at all sites except SSPM-Gran (Figure 6A & 6C). Clusters were evident at scales less than 15 m for SSPM-Meta (mean DBH = 37.1 cm, SE = 4.8 cm) and Teakettle (GoF p-value <0.05), with pulses of aggregation at larger distances including Lost Cannon (30–50 m). Lost Cannon snags were also regularly distributed from 11 m to 45 m (GoF p-value <0.05). Given the low snag densities at the SSPM sites (3.5 to 9.8 ha^−1^; Figure 5), spatial patterns should be interpreted with caution. The bivariate PCF testing for the null hypothesis of random labeling showed a positive correlation of live trees and snags at multiple distances at Lost Cannon (GoF p-value <0.05), while all other sites were not significantly different from random (GoF p-value >0.05; Figure 6B & 6D).

### Patches, Gaps, and Single Trees

**Figure 7:**
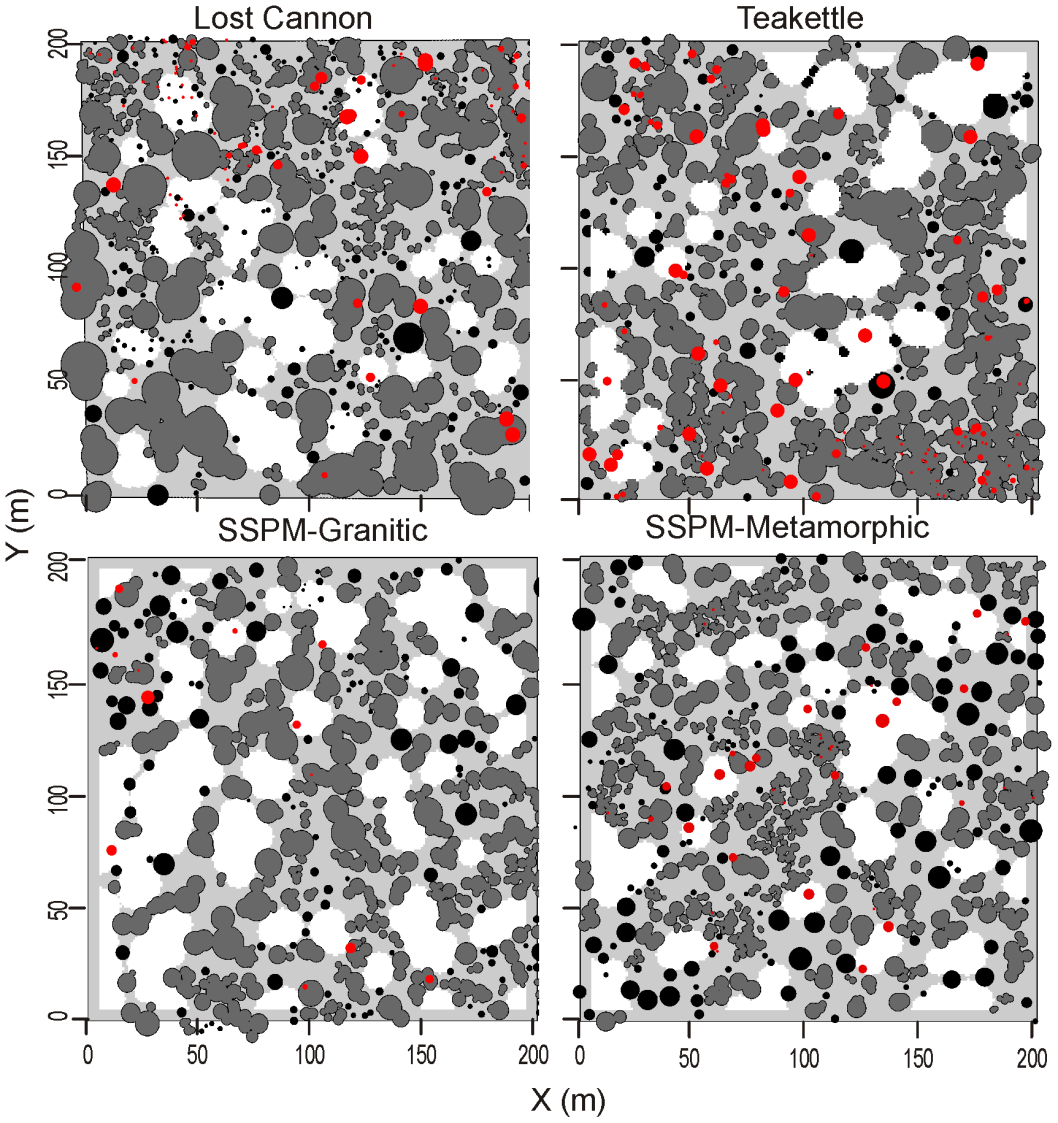
Stem maps of trees (diameters >5 cm) of four old-growth Jeffery pine-mixed conifer forests. Tree patches (gray) and individual trees (black circles) are defined by crown diameters; two or more trees with overlapping crowns define a patch (see Table 3). White areas represent gaps in the forest (see Table 5). Red circles are snags defined by DBH (cm).

**Figure 8:**
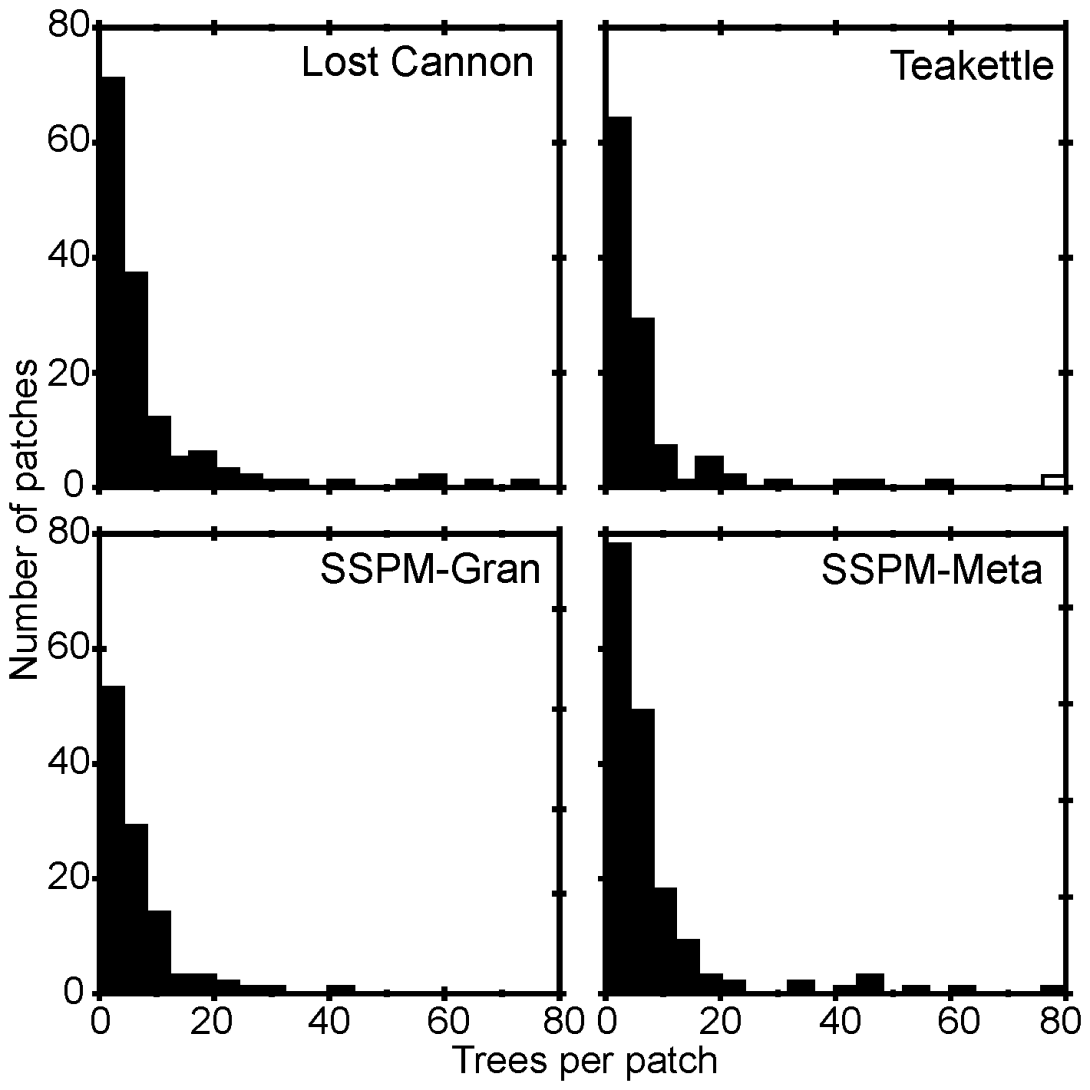
Tree patch distributions in four old-growth Jeffrey pine-mixed conifer forests. At Teakettle, the open bar represents the three largest patches comprising 181 to 211 trees.

**Figure 9:**
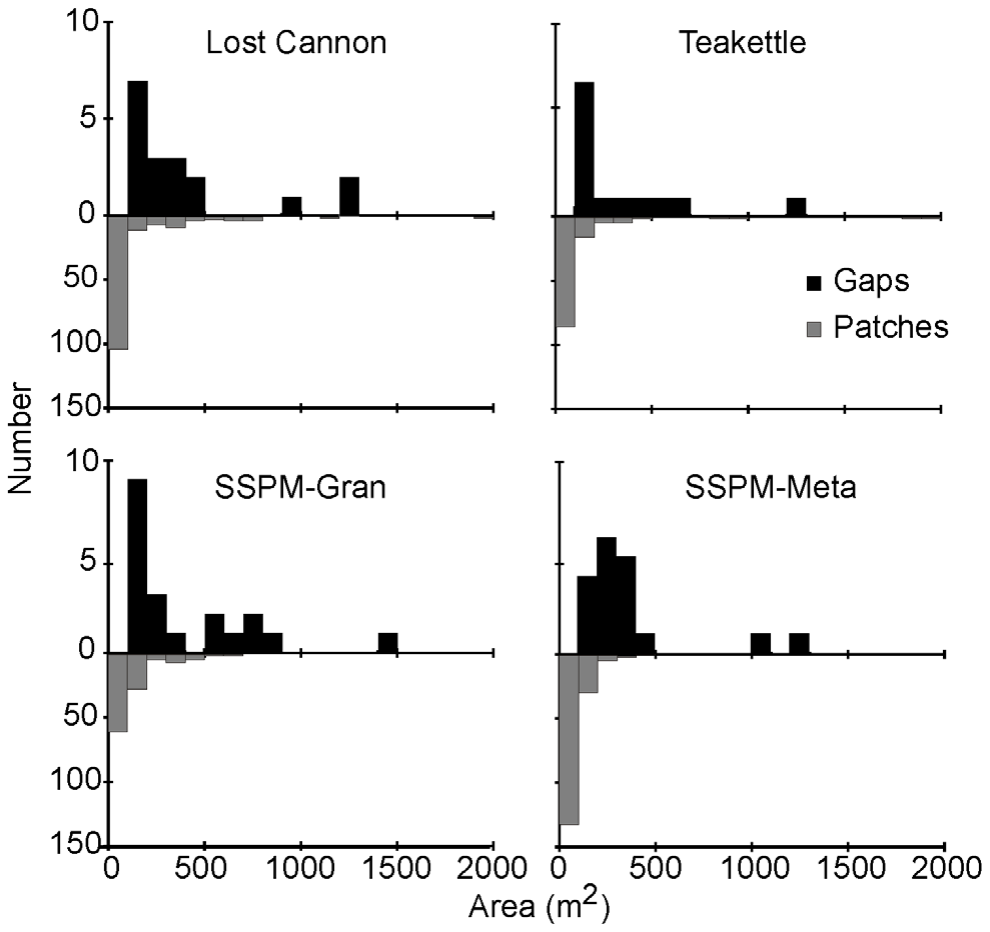
Forest patch and gap area distributions in four old-growth Jeffrey pine-mixed conifer forests. Areas were estimated from four ha stem maps. Not shown is the largest patch at Teakettle with an area of 2589 m^2^.

The majority of live trees (84–93%) were a member of the 534 patches from the four sites (Figure 7). There were overall significant differences in most patch attributes among sites, with trends evident in the pairwise comparisons (Tables 3 & 4). Mean number of trees per patch ranged from six to 11 trees (p-value = 0.753), with three dense patches (181–211 trees) from Teakettle identified as outliers in the model (Figure 8). Among the numerous two-tree patches, accounting for 31% to 36% of the total patches (Table 4), the smallest patch area was 1.4 m^2^ at Lost Cannon and the largest was 174 m^2^ at Teakettle. All of the largest patch areas greater than 0.1 ha were at Lost Cannon and Teakettle (Figure 9). In the smaller trees per patch categories, tree density and patch area were consistently higher at Lost Cannon compared to the other sites, although the proportion of trees in these patches was higher at the SSPM sites. In patches with 10 or more trees this pattern was transposed, where SSPM-Meta had significantly higher tree density and smaller patch area (Table 4).

**Table 3:** Structural attributes of four old-growth Jeffrey pine-mixed conifer forests (patch values represent means (one standard error)).

	Lost Cannon	Teakettle	SSPM-Gran	SSPM-Meta
	Single Tree			
Trees Not in Patches	176	95	101	133
Proportion of Trees(%)	13.2	6.9	14.0	9.7
Mean DBH (cm)	15.6 (1.1)^a^	26.1 (2.7)^b^	29.1 (2.4)^b^	34.3 (2.3)^b^
Basal Area (m^2^ ha^−1^)	2.8^a^	4.1^b^	4.1^b^	6.7^b^
	Patch			
Trees Per Patch	8.1 (1.0)^a^	11.2 (3.0)^a^	5.8 (0.6)^a^	7.4 (0.8)^a^
Tree Density (ha^−1^)	191.4 (17.4)^a^	106.0 (10.9)^bc^	79.3 (6.4)^b^	144.4 (9.4)^ac^
Size (m^2^)	127.6 (20.6)^a^	142.9 (33.3)^ab^	113.3 (12.1)^b^	66.5 (4.5)^a^
Basal Area(m^2^ patch^−1^)	4.0 (0.8)^a^	11.7 (9.3)^b^	3.5 (0.4)^a^	2.1 (0.2)^a^
	Stand			
Density (patchesha^−1^)	35	28.5	25.8	41.5
% Area in Patches	45.7	41.0	30.0	27.7

Within rows, means (one standard error) followed by same letters are not significantly different (*p*>0.05).

**Table 4:** Summary of patch attributes in four old-growth Jeffrey pine-mixed conifer forests.

	Lost Cannon	Teakettle	SSPM-Gran	SSPM-Meta
	2 Trees			
N	45	36	38	54
Proportion of Trees (%)	6.7	5.2	10.5	7.9
Tree Density (stems ha^−1^)	2932.8 (449.4)^a^	1104.8 (286.2)^b^	849.6 (117.7)^b^	1243.4 (174.7)^b^
Basal Area (m^2^ ha^−1^)	48.7 (3.2)^a^	77.0 (6.8)^b^	56.8 (3.9)^ab^	55.2 (3.7)^ab^
Area (m^2^)	18.0 (3.7)^a^	37.6 (6.1)^b^	42.9 (5.0)^b^	37.8 (4.2)^b^
	2–4 Trees			
N	86	73	68	102
Proportion of Trees	17.0	13.9	25.0	20.1
Tree Density	2454.9 (256.0)^a^	1077.4 (153.9)^b^	822.6 (81.8)^b^	1162.3 (111.6)^b^
Basal Area	50.8 (2.5)^a^	86.1 (5.7)^b^	60.7 (2.9)^a^	59.9 (2.8)^a^
Area	23.6 (3.0)^a^	44.4 (5.8)^b^	58.1 (5.5)^b^	48.6 (3.9)^b^
	5–9 Trees			
N	29	24	24	36
Proportion of Trees	13.8	10.5	23.7	17.5
Tree Density	1421.7 (234.6)^ab^	1065.2 (203.7)^ab^	827.1 (141.9)^a^	1549.3 (185.1)^b^
Basal Area	68.2 (5.5)^a^	105.6 (8.0)^b^	65.4 (3.6)^a^	58.4 (3.8)^a^
Area	115.2 (22.2)^ab^	87.6 (11.1)^ab^	143.0 (22.6)^a^	70.9 (9.1)^b^
	10+ Trees			
N	29	18	15	30
Proportion of Trees	56.1	68.7	37.3	52.7
Tree Density	838.6 (109.2)^a^	1006.3 (141.8)^a^	734.2 (116.4)^a^	2243.6 (219.3)^b^
Basal Area	82.5 (3.5)^a^	122.7 (10.1)^b^	73.9 (1.8)^a^	55.6 (3.0)^c^
Area	448.4 (72.8)^a^	615.8 (176.3)^a^	316.2 (43.5)^a^	122.2 (14.6)^b^

Within rows, means (one standard error) followed by same letters are not significantly different among sites (*p*>0.05).

**Figure 10:**
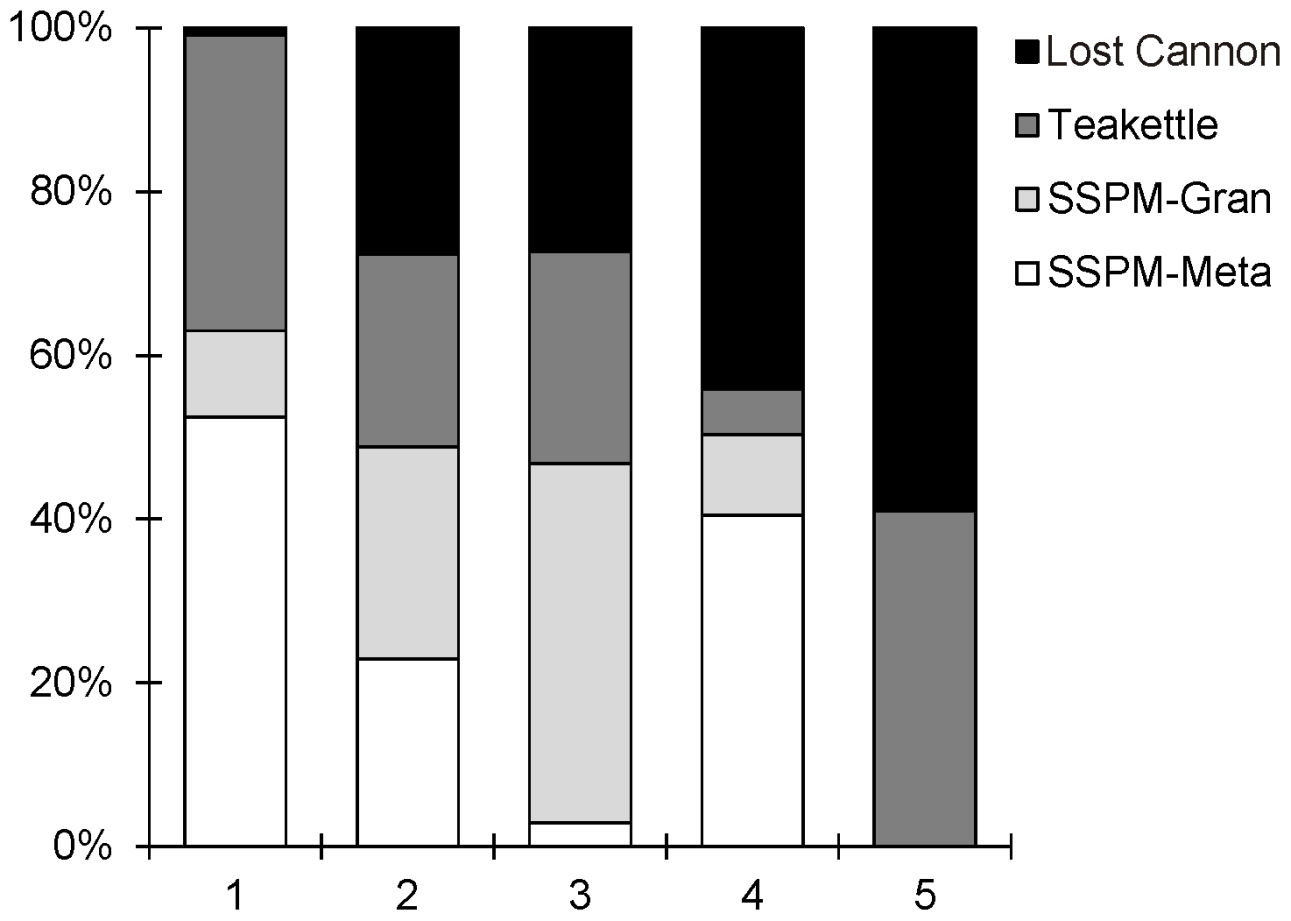
Principal components analysis (bottom right graph) identified several forest structure variables in characterizing 534 tree patches from four stem-mapped plots. Boxplots summarize forest structure variables by groups of patches (top and left graphs) using cluster analysis, which maintained statistical significance (within a boxplot, group number with same superscript are not significantly different; p-value <0.05). Lines inside boxes is the median; boxes show the interquartile range (25–75%) and vertical lines represent the range 10–90%. Width of the boxes is proportional to the square root of the number of patches in the group.

**Figure 11:**
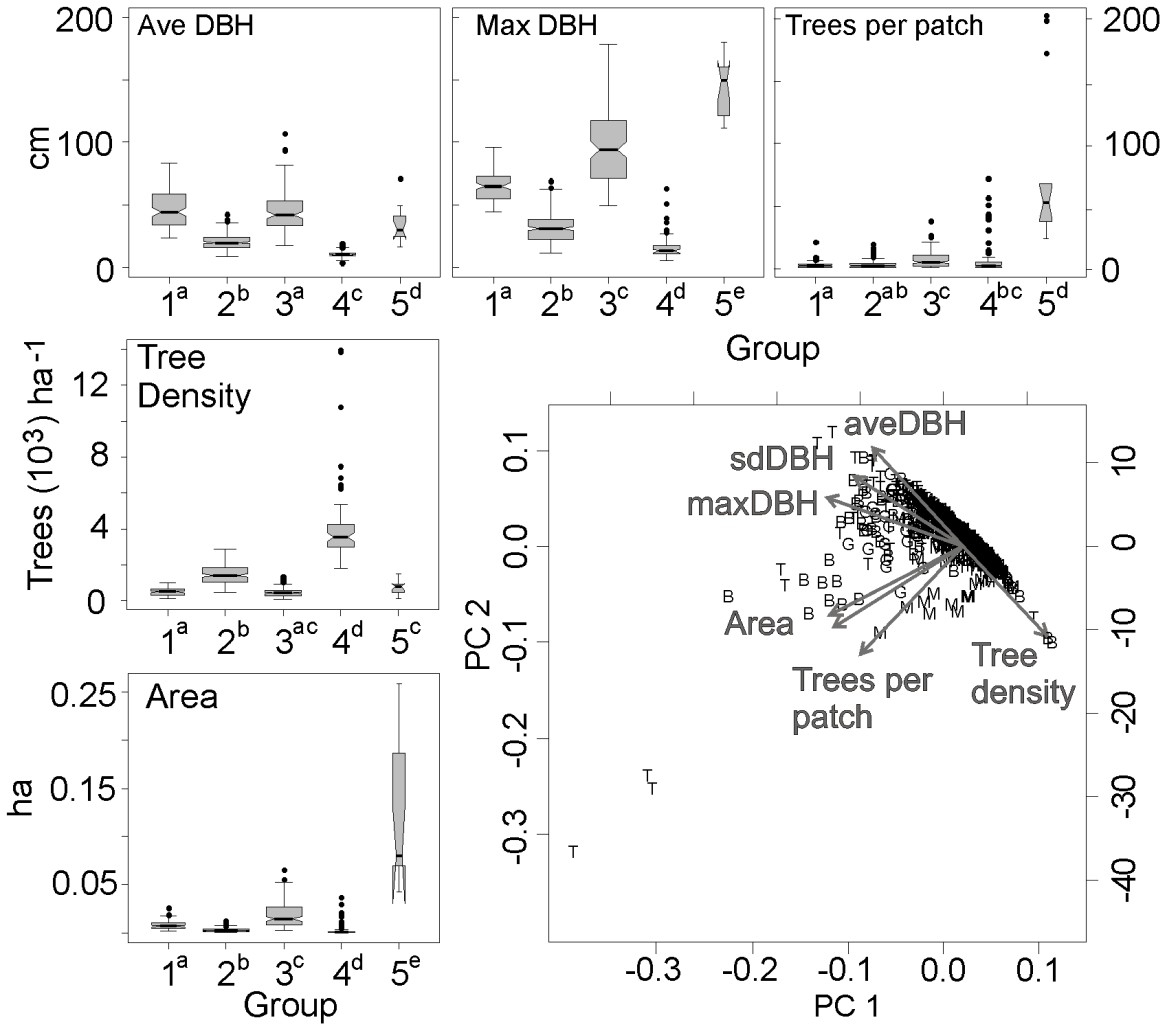
Proportion of 534 tree patches sorted by group number from four old-growth forest plots. A group represents a set of tree patches with similar forest characteristics interpreted using cluster analysis (see Figure 10).

We used the patch structure variables identified in the PCA as inputs in the hierarchical cluster analysis to sort and interpret the 534 tree patches into groups with similar structural attributes. Maximum DBH and patch area were associated with the first axis (PC1), explaining 54.4% of variation in the PCA (Figure 10). Number of trees per patch and mean DBH explained equal variance in the second axis (PC2 = 28.6% of variation). Tree density and standard deviation of the patch mean DBH were associated with the third axis, although PC3 only accounted for an additional 8.3% of the variation. Branch elongation in the cluster dendrogram, implying group stabilization, occurred at five to six groups. Statistical significance (p-value <0.001) was maintained for each variable with five groups (Figure 10). Groups 1 and 3 had similar distributions for most patch variables, in contrast to groups 2 and 4. Low mean tree density, large patch area, and large trees characterized group 5, consisting of outlier patches from Lost Cannon and Teakettle (Figure 11).

Mean diameter for solitary trees was significantly different among sites (p-value <0.001; Table 3), with Lost Cannon being significantly smaller than the rest of the sites (Figure 5). By proportion, Lost Cannon and SSPM-Gran had the highest number of solitary trees and twice as many as Teakettle (Table 3). The average DBH for single trees was higher than the patch average DBH for 68% of the patches at Lost Cannon, 63.5% at Teakettle and SSPM-Gran, and 35.7% at SSPM-Meta. The smallest (113 m^2^) and largest (1413 m^2^) gaps were in SSPM-Gran, which also had the most gaps, the largest range in gap sizes (Figure 9), and the highest percentage of area in gaps. However, there was no significant difference in gap size among sites (p-value = 0.920; Table 5).

**Table 5:** Forest gap characteristics in old-growth Jeffrey pine-mixed conifer forests in California and northwestern Mexico, where summaries were calculated in two ways: all gaps within the four ha study area (with edge gaps), and excluding gaps where more than 10% of the gap area overlapped the 5 m perimeter buffer (without edge gaps).

	Edge Gaps	Numberof Gaps	% Areain Gaps	Mean Gap Size(Std. error) m^2^
Lost Cannon	with	19	18.3	385.6 (81.6)
	without	18	17.7	392.4 (86.0)
Teakettle	with	20	17.3	346.4 (70.7)
	without	13	11.1	341.4 (87.7)
SSPM-Gran	with	29	30.6	421.7 (103.5)
	without	20	19.8	395.3 (73.8)
SSPM-Meta	with	29	22.8	314.7 (50.9)
	without	18	16.4	363.5 (70.8)

## Discussion

Despite the physiographic differences, all four old-growth, Jeffrey pine-mixed conifer forest sites had similar fire return interval summary statistics prior to the onset of fire suppression in their respective jurisdictions. Prior to fire suppression, these forest types supported a fire regime that was characterized by primarily low- to moderate-severity effects with high variability [25], [45], [51]. This type of fire regime is thought to have created very complex spatial patterns both within stands and across landscapes [16], [35], [43], [67]. A recent focus on within-stand spatial patterns identified three salient structural elements: patches of trees, solitary trees, and canopy gaps (reviewed in [35]). They demonstrated a common mosaic structure of these elements typically from <0.4 ha to 4 ha among a variety of forest types. In this study, we found broad similarities in these characteristics reported in other studies in frequent-fire forest types in western US [35], and those discussed therein; [36], [37], [61], [62], yet with distinct differences in patch attributes that were more likely due to site-specific environmental conditions. We hypothesized that at the Sierra Nevada sites would have higher tree densities, fewer and smaller gaps, and less spatial variability due to a century of fire suppression. Teakettle, the most productive of the four sites, and Lost Cannon had higher tree and snag densities, lower percentages of area in gaps, smaller average gap sizes, and lower spatial patchiness in trees, yet they still had many patch and tree attributes that were not significantly different from the comparatively more xeric SSPM sites. The most noteworthy finding was the higher proportion of trees in larger patches and differences in the proportion of patch types (patch groups 1–5 derived from cluster analysis). These differences are likely due to infilling, where in the absence of fire conifer regeneration occurs in canopy gaps merging some tree patches (e.g., [37]), creating highly complex patch types. These findings highlight the complexity of comparing forest patterns across sites where fire in conjunction with many ecosystem processes modifies structural attributes.

We caution that our study has limitations that may affect our results relative to other similar studies. First, using trees larger than 5 cm in diameter gives an incomplete characterization of forest structure by underestimating spatial distributions and patch attributes. Seedling and sapling densities range from 125 stems ha^−1^ to as high as 2247 stems ha^−1^ in similar plots in the SSPM [49] and Teakettle [68], respectively. It is difficult to ascertain the effect of seedlings and saplings on forest structural patterns. Second, the same tree-size classes were used for all sites and do not reflect age classes, especially for SSPM-Meta. Despite size-age relationship differences between sites, we selected size classes that offered a distinction between a century of regeneration with and without fire as a basis for comparisons. Finally, we used an inter-tree distance based on crown radii to delineate tree patches, similar to methods used in Lydersen et al. [37] and Sanchez Meador et al. [62]. Other studies have used a single inter-tree distance of 6 m [35], [36], [61], [69], which is equivalent to a mature Jeffrey pine (50–60 cm DBH) at our sites. A single inter-tree distance value may be operationally simpler [36] but our use of crown radius equations from [63] and others (see methods in Lydersen et al. [37]) approximate when tree crowns actually overlap and thereby may make the results ecologically more relevant.

### Snag Characteristics

Snag characteristics described in this study represent a single representation of conditions, resulting from numerous mortality events from multiple causes, with one exception for the Sierra Nevada sites where fire has not occurred in over a century. Fire is just one of many factors causing tree mortality, both directly through thermal heating and indirectly through injury which can pre-dispose trees to attack by bark beetles (Family Scolytidae), and pathogens (reviewed in [70]). However, these other agents do not need fire to precede them in order to kill trees [40], [71]. North et al. [72] and Innes et al. [73] explained that snag conditions at Teakettle were due primarily to increased stem densities from fire suppression, bark beetle attacks, and drought stress. Snag distribution was described as clustered, which was corroborated in this study, primarily at very small spatial scales (5–10 m). The same causes of mortality would apply at Lost Cannon but a large proportion of snags were small quaking aspen (61%), which may have contributed to the regular spatial distribution and a positive association with live trees.

One significant difference between the Sierra Nevada sites and the SSPM sites is the presence of snags, particularly large snags. The SSPM has very low snag densities, with an annual mortality rate of 0.16% per year in the SSPM-Gran forest type [49]. Reasons for the lower snag densities may include the intact SSPM fire regime [74], [75] that promotes a heterogeneous fire effects pattern [28] where areas within burn perimeters are not burned due to local fuel conditions, maintaining a low recruitment of new snags [76]. Furthermore, snags that are created may be disproportionately small. Three years following a severe drought and wildfire in the SSPM resulted in 22% tree mortality, 36% of these were less than 20 cm DBH [28] and those snags would be consumed in future fires more quickly compared to large snags [77].

### Patches

A cluster of trees with overlapping crowns, varying in size and shape is characteristic of old growth, fire-frequent forests in the western US. Recently studies have reported on these and other stand structural components with the following common elements: trees per patch, patch area, within patch tree size distributions, and species composition [35]–[37]. The number of trees per patch in this study varies greatly, as high as 42–76 trees in SSPM-Meta, but is predominately skewed towards smaller numbers (e.g., 82–86% of patches with 2–9 trees at SSPM). Similarly, patch area consistently reaches a maximum of 0.005 ha for the small trees per patch categories, to 0.2–0.3 ha for even the largest categories at the Sierra Nevada sites. These larger patches are probably mixed aged, with high variability in within patch tree densities, basal areas, tree sizes, and a mixture of shade tolerant and intolerant species [37], [72]. In the absence of fire, stand heterogeneity decreases through a loss of tree patchiness, species diversity, patch growth, and patch expansion into gaps [37], although gaps were still a prominent feature in the Sierra Nevada sites in this study, which were never harvested and have not burned in over a century. At these sites local edaphic conditions (e.g., rock outcrops, shallow soils) may contribute to the persistence of gaps [72], [78].

We reported patches in several categories to facilitate comparisons with other studies and identify potential shortcomings associated with using an intertree distance based on crown radius. Overlapping tree crown projections as the basis of identifying a patch is a direct realization of the influence of the forest canopy on the microenvironmental conditions and processes [51], [68], [79], [80]. While there is no evidence that a 2-tree patch is any different from a 3-tree patch [62], and a substantial number of 2-tree patches were found at all sites, the smallest patch in our study was only 1.4 m^2^. It is not clear what influence small patches may have on microenvironmental processes, but we identify this area as a research need that could help managers focus on patch attributes that positively influence ecosystem structure and function.

Cluster analysis identified several patch types with distinct structural attributes, similar to those described in [35]. These include two common but contrasting types: small patches (less than 0.05 ha) with high densities of mostly small trees; and larger area patches with low tree densities and a large range in tree sizes. These were common at all sites while the last type–low-density patches, some large trees and high variability in area–was uncommon and occurred only at the Sierra Nevada sites. These differences in proportion of patch types and trees per patch categories between the two regions are the defining characteristic of the effect of both wildfire [28], [81]–[83] and prescribed fire [82], [84] as a process that modifies structural characteristics and maintains heterogeneity at small spatial scales.

Comparisons in structural attributes between the two SSPM forest sites are noteworthy. Separated by only two km, these sites have analogous elevations, fire regimes [25], and climate [26]. They also had similar proportions of patches (61–64%) in the smaller patch categories (≤4 trees per patch) and patch size, density, and basal area were not significantly different. In contrast, SSPM-Meta supports more trees in large patch types, larger solitary trees, significantly higher density, lower basal area, and smaller patch area. Geochemical properties of SSPM-Gran and SSPM-Meta are distinct and the likely basis for the differences seen here. Soils in the SSPM-Gran are typical of similar forests in the Sierra Nevada [50] and have substantially coarser texture, more phosphorus, but less magnesium and potassium [49] compared to SSPM-Meta (Stephens, unpublished data). Few other studies have identified unique structural attributes that are likely due to the strong influence of soils (e.g., [61]) and illustrate the need for this type of information.

### Canopy Gaps

The range in gap areas of 0.006 to 0.289 ha were at the lower end of what has been reported in similar forest types that quantified gap characteristics (reviewed in [35]–[37]). Differences are due to gap definitions, methods used to quantify gaps, and stand history. In our study, we used the PatchMorph tool in arcGIS, which requires user-defined gap dimensions. Our thresholds were chosen to limit small and highly convoluted shapes, which was the same approach used by [37]. Given the fact that our study sites can be considered reference sites for similar forest types throughout CNB, the choice to capture simpler gap shapes may be particularly relevant for forest restoration. Promoting pine growth and regeneration are common objectives in forests where fire suppression has increased densities of small trees and shifted composition towards shade-tolerant species [18], [19], [29], [37], [42]. Silvicultural studies have identified canopy openings ranging 0.02–0.1 ha as appropriate gap sizes for promoting establishment and growth of shade-intolerant tree species (e.g., [85]–[88]). However, we recognize that a more realistic feature is gaps with convoluted shapes [36], [37] and flexibility in gap definitions allows management-specific questions to be addressed.

There was no difference in average gap size among the four sites despite the long fire-free period in the Sierra Nevada sites. This is noteworthy for a couple of reasons. First, Teakettle is a far more productive site with a higher proportion of shade-tolerant species compared to the other sites. These two factors would lead to the assumption that in the absence of fire, gaps would become occupied by trees, as was demonstrated by Lydersen et al. [37]. Despite overall increases in tree density and shifted species composition towards greater proportions of shade-tolerants in the last 100 years at Teakettle, the infilling of gaps does not appear to have been substantial [72]. Historical photographic evidence combined with the lack of successful establishment of planted saplings in open areas suggests that some gaps at Teakettle may be edaphically controlled [78], and therefore fairly fixed in space [68], [72], [89]. Perhaps the same explanation applies to persistence of gaps at Lost Cannon. Another explanation of why gaps persist in the two Sierra Nevada sites despite the long fire-free period could be related to lack of timber harvesting at both sites, which left the large tree component intact. This is in contrast to sites studied by Lydersen et al. [37], which were harvested in the early 20^th^ century, primarily focused on removal of larger diameter trees. This loss of the large tree component fundamentally altered forest stand conditions by opening up a substantial amount of growing space [67], [90]. More research may be necessary to better understand the effects associated with large tree removal, and the potential interactions with fire suppression/exclusion.

### Single Trees

Solitary trees are a common structural component of fire-frequent forests [91], and the size and proportion of the total trees within a stand appear to vary greatly (reviewed in [35]). Even in the SSPM where fire regimes were still intact into the late 1960s, the proportion of single trees (10–14%) was similar to the fire-suppressed Sierra Nevada sites (7–13%). At a 1929 stem mapped reference site in the Sierra Nevada [37], the proportion of single trees in a mixed-conifer forest was even smaller at 6%. It is worth noting that the minimum DBH in their study was 10 cm, while for this study it was 5 cm. Availability of resources, edaphic conditions, species composition, past logging, and stand development stage influence tree-tree separation distances [92], but additional research is needed to determine the factors that dictate patterns in forests [35].

Average diameters of solitary trees were not always higher than patch average diameters, as has been reported in other forests [62], [93]. The largest percentage was from SSPM-Meta, which also had the strongest segregation in spatial distributions among size classes. Larger trees produce more fine fuels, which will burn more frequently and limit seedling recruitment beneath their canopies [28], perpetuating spatial segregation [53], [61], [72], [75], [94]. This effect may be ameliorated in multi-species, multi-aged stands where varying resources may be utilized differently and where resources are not as limited [37], [61].

### Management Implications

In fire-dependent forest ecosystems, a decrease in fire frequency due to our efficiency in extinguishing wildfires has resulted in forest structure and surface fuels conditions that increase the potential for uncharacteristic fire sizes and effects [95]. Beyond the CNB, the impacts of fire suppression and exclusion policies on ecosystem health exist in the MB (e.g., [96], [97]), Australia (e.g., [98]), and elsewhere; indeed, this is a global issue. Reference forests provide insight into how structural components may vary under an intact fire regime, and provide a baseline of information from which to guide restoration and fuel reduction treatments. Unfortunately, many of these regions have very few, if any, intact forests that can serve this purpose [4]. The western US has the benefit of historical information and the few remaining old-growth forests, including the northwestern Mexico sites used in this study, to use as ecological models. Recreating stand conditions based on reference site information should not be the sole objective. Rather, conditions that allow ecosystem processes to reestablish old-growth conditions and increase resiliency are a higher priority [38], [99]. This will require flexible management and regulation oversight as ecological processes realign, especially under potentially novel future climate conditions [100], [101].

Restoring ecosystems through prescribed fire and fuel reduction treatments has been effective at reducing fire behavior of wildfires, decreasing tree mortality, and providing defensible space for communities and opportunities for suppression operations [97], [102]–[105]. However many past fuel treatments emphasized homogenous forest structures that were outside of past ecological conditions [42]. Our effort with this study was to quantify more complex structural and spatial components that can be effectively communicated to managers; tailoring restoration treatments towards these conditions will increase heterogeneity by creating a range of forest conditions based on distributions of patch, gap, and single tree attributes [35]. An example of this is provided by North et al. [89] comparing spatial patterns of reconstructed reference forest conditions to the same forest following several fuel reduction treatments. This highlights the need to consider whether proposed treatments will create or maintain desired structural attributes. Several other studies have incorporated variable densities and structure into silvicultural treatment prescriptions, with contemplations on the difficulty in its implementation [36], [64], [106]. Once treatments have been implemented across multiple stands, repeated prescribed fires, or managed wildfires where feasible, are critical to maintaining key ecosystem processes and heterogeneous structure and composition [89], [107].

## Supporting Information

Figure S1 **The pattern of live adult trees (DBH >25 cm) was contrasted to the null model of complete spatial randomness (CSR), using univariate Ripley’s K (** ***L(r)*** **) and pair correlation function (g** ***(r)*** **).** Approximately 95% simulation envelopes (grey shaded areas) were constructed with 199 Monte Carlo simulations of the CSR model. Spatial aggregation is indicated by the large-scale departure of the observed values (solid black lines) from CSR at Lost Cannon and Teakettle, but not at the SSPM sites.
(TIF)

Methods S1 **Local vs. large scale heterogeneity in point patterns** [**108**] **.**
(DOCX)
