# Supplementary figures and images for: Contrasting Spatial Patterns in Active-Fire and Fire-Suppressed Mediterranean Climate Old-Growth Mixed Conifer Forests
Source: PLoS One. 2014 Feb 20;9(2):e88985. doi: 10.1371/journal.pone.0088985 (PMC3930671; doi:10.1371/journal.pone.0088985)

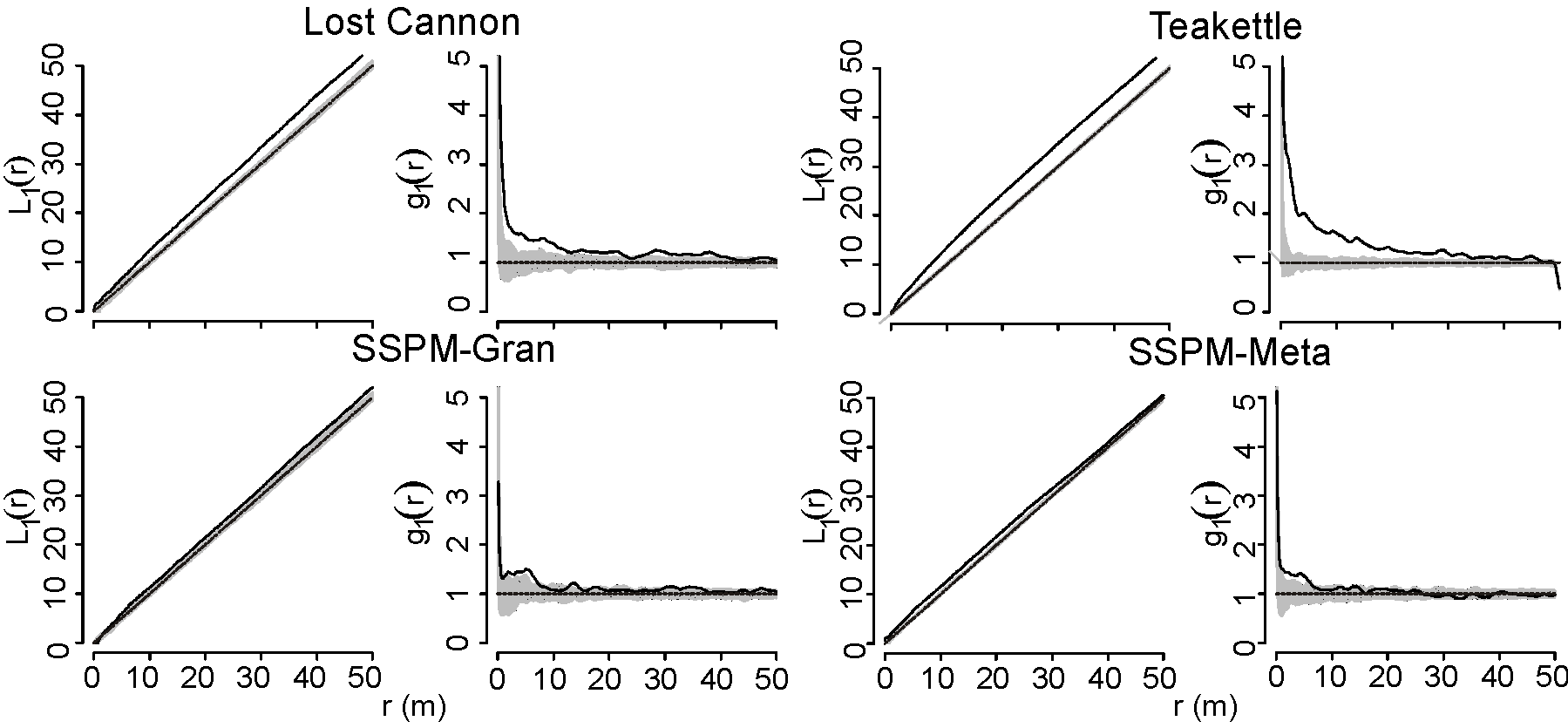

Supplement: Figure S1 — The pattern of live adult trees (DBH >25 cm) was contrasted to the null model of complete spatial randomness (CSR), using univariate Ripley’s K ( L(r) ) and pair correlation function (g (r) ). Approximately 95% simulation envelopes (grey shaded areas) were constructed with 199 Monte Carlo simulations of the CSR model. Spatial aggregation is indicated by the large-scale departure of the observed values (solid black lines) from CSR at Lost Cannon and Teakettle, but not at the SSPM sites. (TIF) [file pone.0088985.s001.tif]
