# Supplementary material for: Contrasting Spatial Patterns in Active-Fire and Fire-Suppressed Mediterranean Climate Old-Growth Mixed Conifer Forests
Source: PLoS One. 2014 Feb 20;9(2):e88985. doi: 10.1371/journal.pone.0088985 (PMC3930671; doi:10.1371/journal.pone.0088985)
Supplement: Methods S1 — Local vs. large scale heterogeneity in point patterns [108] . (DOCX) [file pone.0088985.s002.docx]

**Methods S1. Local vs. large scale heterogeneity in point patterns.**

Identification of local plant-plant interactions (second order effects) may be confounded by larger scale environmental heterogeneity (first order effects; [108]). While we intended to minimize any environmental gradients across the plots, observations of Figure 1 indicate heterogeneity may be present. To test the hypothesis of environmental homogeneity we evaluated univariate distributions of adult trees (DBH > 25 cm) using the two complementary statistics: pair correlation function *g(r)* and Ripley’s *K (r)* (reported in linearized form *L* *(r)*). Following the arguments in Getzin et al. [108], evidence of large scale heterogeneity is evident when clustering of adult trees is present in *L(r)* and *g(r)* at scales beyond tree-tree interactions (i.e., r > 20 m since the largest crown radius is 10.7 m). Any larger scale variations in habitat quality that influence tree establishment should be expressed in the distribution of adult trees. In these cases the inhomogeneous pair correlation function should be used with a non-parametric estimate of the intensity function based on the Epanechnikov kernel [56] of tree distributions and a bandwidth following Stoyan and Stoyan [55].

The pattern of live adult trees (DBH > 25 cm) was contrasted to the null model of complete spatial randomness (CSR), using univariate Ripley’s K (*L(r)*) and pair correlation function (g*(r)*) (Figure S1). Approximately 95 % simulation envelopes (grey shaded areas) were constructed with 199 Monte Carlo simulations of the CSR model. Spatial aggregation is indicated by the large-scale departure of the observed values (solid black lines) from CSR at Lost Cannon and Teakettle, but not at the SSPM sites.

These contrasting results and examination of Figure 1 provide evidence of large-scale heterogeneous environmental conditions at Lost Cannon and Teakettle.
